# Correlation Analysis of Surface and Physical Properties of Ophthalmic Lenses Containing Nanoparticles

**DOI:** 10.3390/mi14101883

**Published:** 2023-09-30

**Authors:** Su-Mi Shin, Hye-In Park, A-Young Sung

**Affiliations:** Department of Optometry & Vision Science, Daegu Catholic University, Gyeongsan 38430, Republic of Korea; soomi8712@naver.com (S.-M.S.); gpdls4731@naver.com (H.-I.P.)

**Keywords:** contact lens, physical properties, surface properties, antimicrobial properties, gold nanoparticle

## Abstract

Since contact lenses directly contact the cornea, the surface roughness of the lens may cause various side effects. In addition, gold nanoparticles can realize a variety of colors and characteristics depending on their shape and size. In this study, the surface roughness of tinted lenses containing gold nanoparticles of various sizes was analyzed using atomic force microscopy (AFM) at aspect ratio(surface to volume ratio) ranging from 1:1 to 1:10. The characteristics of the lenses were then confirmed. As a result, tinted lenses with different colors depending on the size of the gold nanoparticles were manufactured. The surface roughness of the lens decreased with increasing size of the gold nanoparticles. However, at aspect ratio of 1:10, increase in surface roughness was observed. In addition, it was confirmed that the wettability and antibacterial properties of the lens had the same effect according to the average surface roughness value. Therefore, it was confirmed that the addition of gold nanoparticles reduced the surface roughness of the lens, which had a great effect on properties such as wettability and antimicrobial properties of the lens. The produced copolymer controls the surface roughness of the lens, and thus it is judged that it can be used as a material for various ophthalmology applications.

## 1. Introduction

Compared to spectacle lenses, contact lenses have the advantage of having a wider field of view and fewer optical aberrations [1]. They are used not only for vision correction but also for cosmetic purposes since they do not cover the face. In particular, lenses with colors are very popular among young women because they can change their image. For contact lenses, products with excellent wearing comfort are preferred, and wearing comfort is greatly influenced by water content, oxygen permeability, and wettability [2]. Silicone hydrogel material has high oxygen permeability and is currently widely used in contact lenses [3]. However, many studies are underway to improve the disadvantages of silicone materials such as low water content and low wettability [4,5,6]. In addition, contact lenses are medical devices that are worn directly on the cornea, and may cause problems and eye diseases such as the introduction of foreign matter, keratitis, and conjunctivitis. As a result of the COVID-19 pandemic, interest in antibacterial properties is gradually increasing [7]. Bacterial infection associated with contact lenses is related to surface properties such as the wettability and surface roughness of the lens [8,9]. In particular, nano gold has excellent antibacterial properties and is currently being used in a variety of fields. Nano gold has many characteristics depending on the particle size, so it is applied by adjusting the size of various particles through synthesis. Previously, numerous studies on coloring effect, antibacterial coating, and color vision correction have been conducted by applying nano gold to contact lenses [10,11,12].

In this study, colored contact lenses were prepared by applying gold nanoparticles with various particle ratios to silicon hydrogel materials. The basic physical properties and surface properties of the manufactured lens were analyzed. In addition, this study aims to confirm the correlation between physical properties through the antibacterial properties of nano gold.

## 2. Materials and Methods

### 2.1. Reagents and Materials

**Figure 1:**
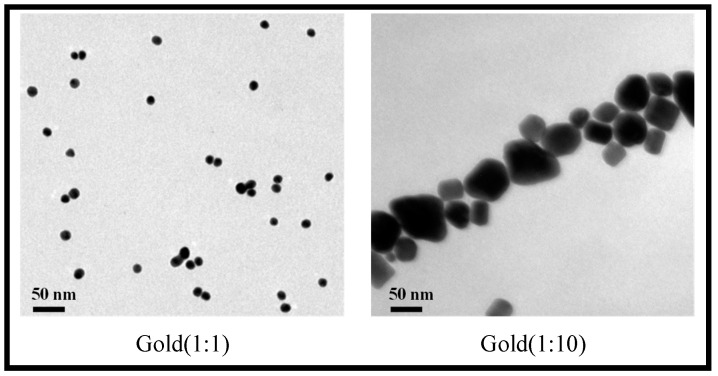
TEM image of gold nanoparticles with different aspect ratios.

The materials in this study were synthetic silicone monomer (SID-OH) with a hydroxy attached structure to polydimethylsiloane, N,N-dimethylacetamide (DMA), ethylene glycol dimethacrylate (EGDMA) as a crosslinking agent, and 2-hydroxy-2-methylpropiophenone (2H2M) as a photoinitiator. All reagents used were Sigma Aldrich products. In addition, gold nanoparticles of various sizes synthesized at an aspect ratio of 1:1 to 1:10 were used as additives, and products from Fine Nano Co., Ltd. (Suwon, Republic of Korea) were used. The Transmission Electron Microscope (TEM) image of the gold nanoparticles used in this study is shown in Figure 1.

### 2.2. Polymerization

In this study, SID-OH, DMA, EGDMA, and 2H2M were copolymerized as basic combinations, and gold nanoparticles with different particle sizes were added to the basic combinations and copolymerized. Each monomer was dispersed in vortex and ultrasonic waves and then photopolymerized. For photopolymerization, a UV curing system (FJ100, Phoseon Technology, Hillsboro, OR, USA), which is a photopolymerization device, was used, and UV was irradiated at a wavelength of 365 nm to polymerize.

A contact lens made of the basic combination was named REF. In addition, contact lenses with gold nanoparticles used as additives were named G1, G3, G5, G7, and G10 according to the size of the particles. The mixing ratio of the manufactured lenses is presented in Table 1.

### 2.3. Instruments and Analysis

All the manufactured lenses were hydrated in 0.9% sodium chloride physiological saline for 24 h and then their physical properties were measured. The manufactured lenses were measured for physical properties such as refractive index, water content, and tensile strength, and surface area properties such as contact angle, atomic force microscope (AFM), and scanning electron microscope (SEM). The contact angle evaluated the wettability of the lens surface using the sessile drop method. AFM measured the surface roughness of the lens using a cantilever probe. SEM scanned the surface of the lens using an electron beam to confirm the gold nanoparticles added to the lens. In addition, the antibacterial property of the lens was confirmed with an antibacterial test. The antibacterial test used 3M Petrifilm^TM^ (3M, St. Paul, MN, USA) a dry medium film, and confirmed the antibacterial properties of the lens against *Escherichia coli* and *Staphylococcus aureus*. All the results presented in this study were averaged by repeating measurements five times per sample to increase accuracy.

## 3. Results and Discussion

**Figure 2:**
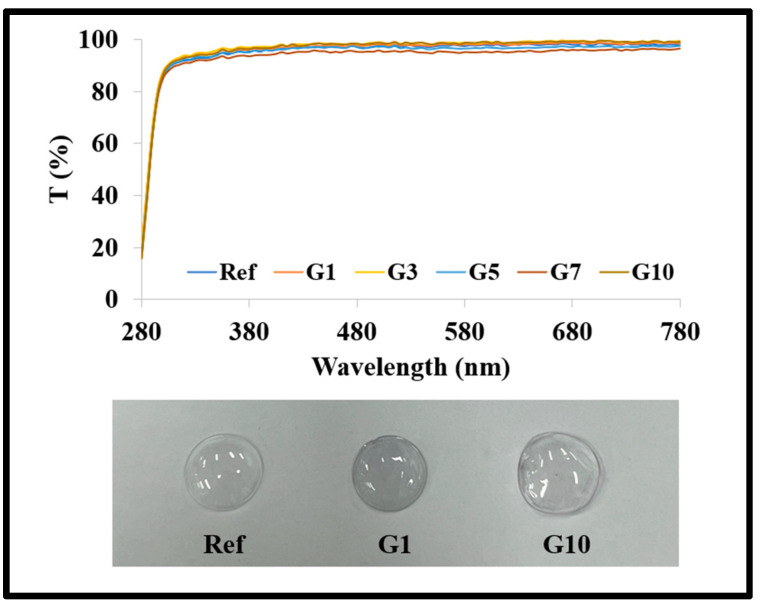
Produced lens and spectral transmittance of samples.

All prepared lenses showed over 90% in the visible transmission. In addition, visually the color of the manufactured lenses gradually changed as the aspect ratio of the gold nanoparticles increased. The spectral transmittance graph and the prepared lens are presented in Figure 2, respectively.

### 3.1. Physical Property

**Figure 3:**
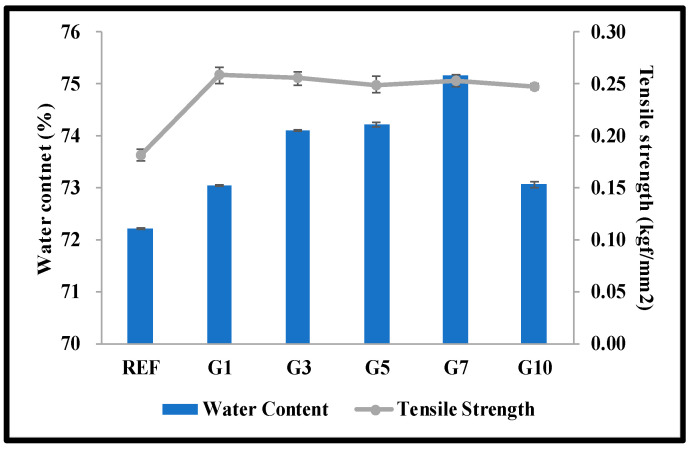
Water content and tensile strength of samples.

In order to confirm the basic physical properties of the manufactured lens, the refractive index, water content, and tensile strength were measured. As a result of measuring the physical properties of REF manufactured with the basic combination, the refractive index was 1.3757, the water content was 72.21%, and the tensile strength was 0.181 kgf/mm^2^. In addition, the lens with gold nanoparticles added to the basic combination was measured by comparing the basic characteristics according to the size of the gold nanoparticles. As a result of measuring the refractive index, as the size of the gold nanoparticles increased; this appeared to be 1.3755 to 1.3756, showing no significant change. However, the water content was 73.04 to 75.15% in G1 to G7 and 73.06% in G10. As the aspect ratio of the gold nanoparticles increased, the water content slightly increased, and the lens (G10) to which the gold nanoparticles were added with a size of 1:10 showed a slight decrease in water content. In addition, the tensile strength was 0.247 to 0.258 kgf/mm^2^ regardless of the size of the gold nanoparticles, and it was confirmed that the tensile strength was improved by the addition of gold nanoparticles. This shows similar results to previous research [13], and it is thought that the tensile strength of the lens was improved due to the characteristics of gold nanoparticles. The graphs of water content and tensile strength of the manufactured lenses are presented in Figure 3.

### 3.2. Surface Analysis

**Figure 4:**
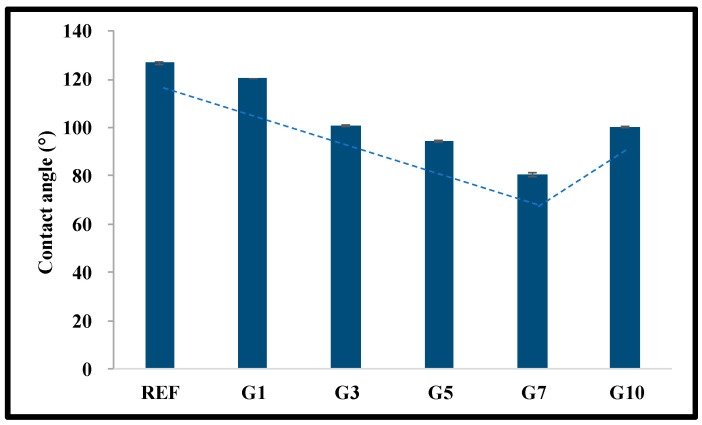
Contact angle of samples.

To analyze the surface of the manufactured lens, contact angle, AFM, and SEM were measured. The contact angle was measured to confirm the wettability of the manufactured lens; the results show that REF was 126.74°, G1 to G7 were 120.47 to 80.56°, and G10 was 100.50°. As the aspect ratio of the gold nanoparticles increased, the wettability was improved due to the lower contact angle, but the contact angle of the lens with of 1:10 gold nanoparticles slightly increased, similar to the result of the water content. The contact angle measurement graph and image are presented in Figure 4.

**Figure 5:**
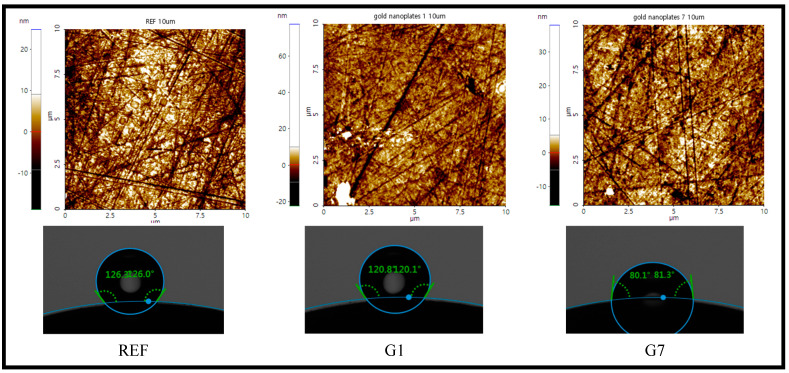
AFM and contact angle images of samples.

AFM was measured to confirm the surface roughness of the manufactured lens. As a result of the measurement, the surface roughness value (Ra) of the lens was found to be REF 3.8 μm, G1 3.1 μm, G3 2.3 μm, G5 2.1 μm, G7 2.0 μm, and G10 2.7 μm, respectively. The measured values of AFM appeared similar to the result values of water content and contact angle. It is believed that the surface roughness of the lens affects the contact angle, and several studies have reported that the surface roughness of the material is related to the contact angle [14,15]. AFM and contact angle images are presented in Figure 5.

**Figure 6:**
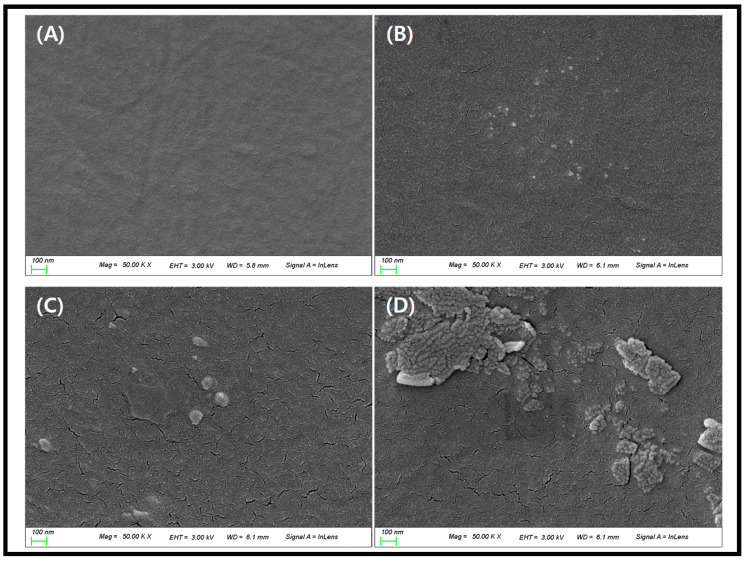
SEM images of samples. (**A**) REF, (**B**) G1, (**C**) G5, (**D**) G10.

In addition, SEM was measured to confirm the surface of the lens to which the synthesized gold nanoparticles were added at an aspect ratio of 1:1 to 1:10. As a result of the measurement, REF without gold nanoparticles was confirmed to have a smooth surface with nothing (A). On the other hand, in the case of the sample with gold nanoparticles added, the particle size of G1 was about 10 nm to 20 nm (B), G5 was about 50 to 100 nm (C), and G10 was about 100 to 200 nm (D). It was confirmed that the gold nanoparticles synthesized at various sizes were well distributed on the lens surface. The SEM measurement image is presented in Figure 6.

### 3.3. Antibacterial Property

**Figure 7:**
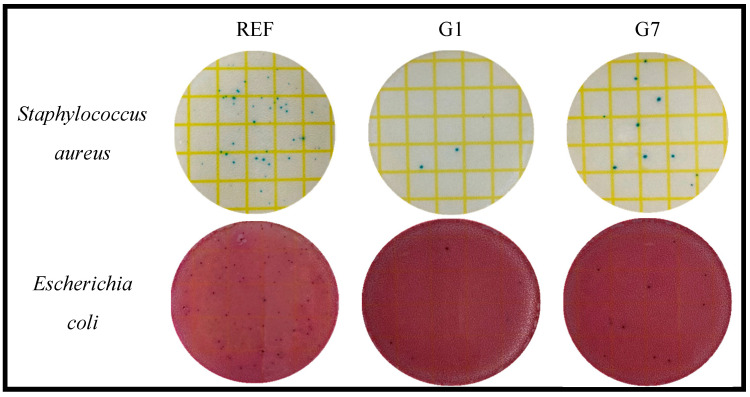
Antimicrobial property of samples.

In order to confirm the antibacterial properties of the prepared lenses, their effects against Staphylococcus aureus and Escherichia coli were confirmed using 3M Petrifilm^TM^, a dry film medium. For the antibacterial test, the prepared lens and physiological saline were added and shaken, then 1 mL of the solution was taken and smeared on 3M Petrifilm^TM^, a dry media film. The plated dry film medium was incubated for about 24 h in an incubator at 35 ± 1 °C, and the antimicrobial effect against each microorganism was confirmed. As a result of the measurement, it was confirmed that numerous microorganisms grew in the medium of REF, which was a control group. On the other hand, all the samples to which the gold nanoparticles were added had excellent antibacterial properties regardless of the type of microorganism. In particular, the antibacterial properties were the highest in the G1 group and slightly lower in the G7 group. The antibacterial properties were inversely proportional to the surface roughness values of AFM. Except for REF, the antibacterial properties of gold nanoparticles are related to the surface roughness of the lens [9,16], and it is believed that the surface roughness interferes with the attachment of microorganisms and thus has a difference in antibacterial properties. The results of antibacterial activity against Staphylococcus aureus and Escherichia coli are presented in Figure 7.

## 4. Conclusions

The contact lenses manufactured in this study were photopolymerized by adding gold nanoparticles with various particle sizes with an aspect ratio of 1:1 to 1:10 to a silicone hydrogel material. The water content and wettability of the lens gradually increased as the particle size of the gold nanoparticles increased, and then decreased again at an aspect ratio of 1:10. In addition, it was shown that the surface roughness value of AFM decreases as the particle size increases, and antibacterial activity decreases accordingly. Therefore, gold nanoparticles with various aspect ratios showed the highest physical properties at an aspect ratio of 1:7. In addition, it was confirmed that physical properties affect surface properties, and, at the same time, surface properties affect antibacterial properties. It is thought that the characteristics of contact lenses can affect each other to compensate for the disadvantages of lens materials.

## Figures and Tables

**Table 1:** Percent compositions of samples. (unit: wt%).

	SID	DMA	EGDMA	2H2M	Gold (1:1) NPs *	Gold (1:3) NPs *	Gold (1:5) NPs *	Gold (1:7) NPs *	Gold (1:10) NPs *
Ref	32.94	65.87	0.99	0.20	-	-	-	-	-
G1	32.77	65.55	0.98	0.20	0.5	-	-	-	-
G3	32.77	65.55	0.98	0.20	-	0.5			
G5	32.77	65.55	0.98	0.20	-	-	0.5	-	-
G7	32.77	65.55	0.98	0.20	-	-	-	0.5	-
G10	32.77	65.55	0.98	0.20	-	-	-	-	0.5

* NPs: Nanoparticles.

## Data Availability

Not applicable.
